# Can RNA-Seq Resolve the Rapid Radiation of Advanced Moths and Butterflies (Hexapoda: Lepidoptera: Apoditrysia)? An Exploratory Study

**DOI:** 10.1371/journal.pone.0082615

**Published:** 2013-12-04

**Authors:** Adam L. Bazinet, Michael P. Cummings, Kim T. Mitter, Charles W. Mitter

**Affiliations:** 1 Laboratory of Molecular Evolution, Center for Bioinformatics and Computational Biology, University of Maryland, College Park, Maryland, United States of America; 2 Department of Entomology, University of Maryland, College Park, Maryland, United States of America; Institut National de la Recherche Agronomique (INRA),, France

## Abstract

Recent molecular phylogenetic studies of the insect order Lepidoptera have robustly resolved family-level divergences within most superfamilies, and most divergences among the relatively species-poor early-arising superfamilies. In sharp contrast, relationships among the superfamilies of more advanced moths and butterflies that comprise the mega-diverse clade Apoditrysia (ca. 145,000 spp.) remain mostly poorly supported. This uncertainty, in turn, limits our ability to discern the origins, ages and evolutionary consequences of traits hypothesized to promote the spectacular diversification of Apoditrysia. Low support along the apoditrysian “backbone” probably reflects rapid diversification. If so, it may be feasible to strengthen resolution by radically increasing the gene sample, but case studies have been few. We explored the potential of next-generation sequencing to conclusively resolve apoditrysian relationships. We used transcriptome RNA-Seq to generate 1579 putatively orthologous gene sequences across a broad sample of 40 apoditrysians plus four outgroups, to which we added two taxa from previously published data. Phylogenetic analysis of a 46-taxon, 741-gene matrix, resulting from a strict filter that eliminated ortholog groups containing any apparent paralogs, yielded dramatic overall increase in bootstrap support for deeper nodes within Apoditrysia as compared to results from previous and concurrent 19-gene analyses. High support was restricted mainly to the huge subclade Obtectomera broadly defined, in which 11 of 12 nodes subtending multiple superfamilies had bootstrap support of 100%. The strongly supported nodes showed little conflict with groupings from previous studies, and were little affected by changes in taxon sampling, suggesting that they reflect true signal rather than artifacts of massive gene sampling. In contrast, strong support was seen at only 2 of 11 deeper nodes among the “lower”, non-obtectomeran apoditrysians. These represent a much harder phylogenetic problem, for which one path to resolution might include further increase in gene sampling, together with improved orthology assignments.

## Introduction

The insect order Lepidoptera (moths and butterflies; >157,000 spp.; [1]) is arguably the largest single radiation of plant-feeding insects. A prominent element of terrestrial ecosystems, Lepidoptera function as herbivores, pollinators and prey, with substantial impact on humans. Highly destructive as agricultural pests, they have also become icons for environmental conservation, and supply food and fiber to multiple societies [2]. And, they provide important model systems for studies of genetics, physiology, development, and many aspects of ecology and evolutionary biology [3], including the question of why herbivorous insects, 25% of earth’s known species, are so species-rich [4-6].

A robust phylogenetic framework is essential for all attempts to understand the diversity, adaptations and ecological roles of Lepidoptera. The past decade has seen tremendous advances in our understanding of lepidopteran phylogeny at all levels. Molecular data have proven especially powerful for defining superfamilies and relationships within them. In a remarkable burst of community progress, robust molecular phylogenies for nearly all of the major superfamilies (those containing hundreds to thousands of species), combined with review of the morphological evidence, have been published in the past few years or will be forthcoming shortly. Recent examples (not an exhaustive list) include studies of Bombycoidea [7], Gelechioidea [8], Geometroidea [9-11], Gracillarioidea [12], Noctuoidea [13,14], Papilionoidea [15], Pyraloidea [16], Tortricoidea [17], and Yponomeutoidea [18]. In all of these superfamilies, a majority of the major divergences (at least) seem credibly established, though important uncertainties remain. Progress is also now rapid at more subordinate levels.

**Figure 1:**
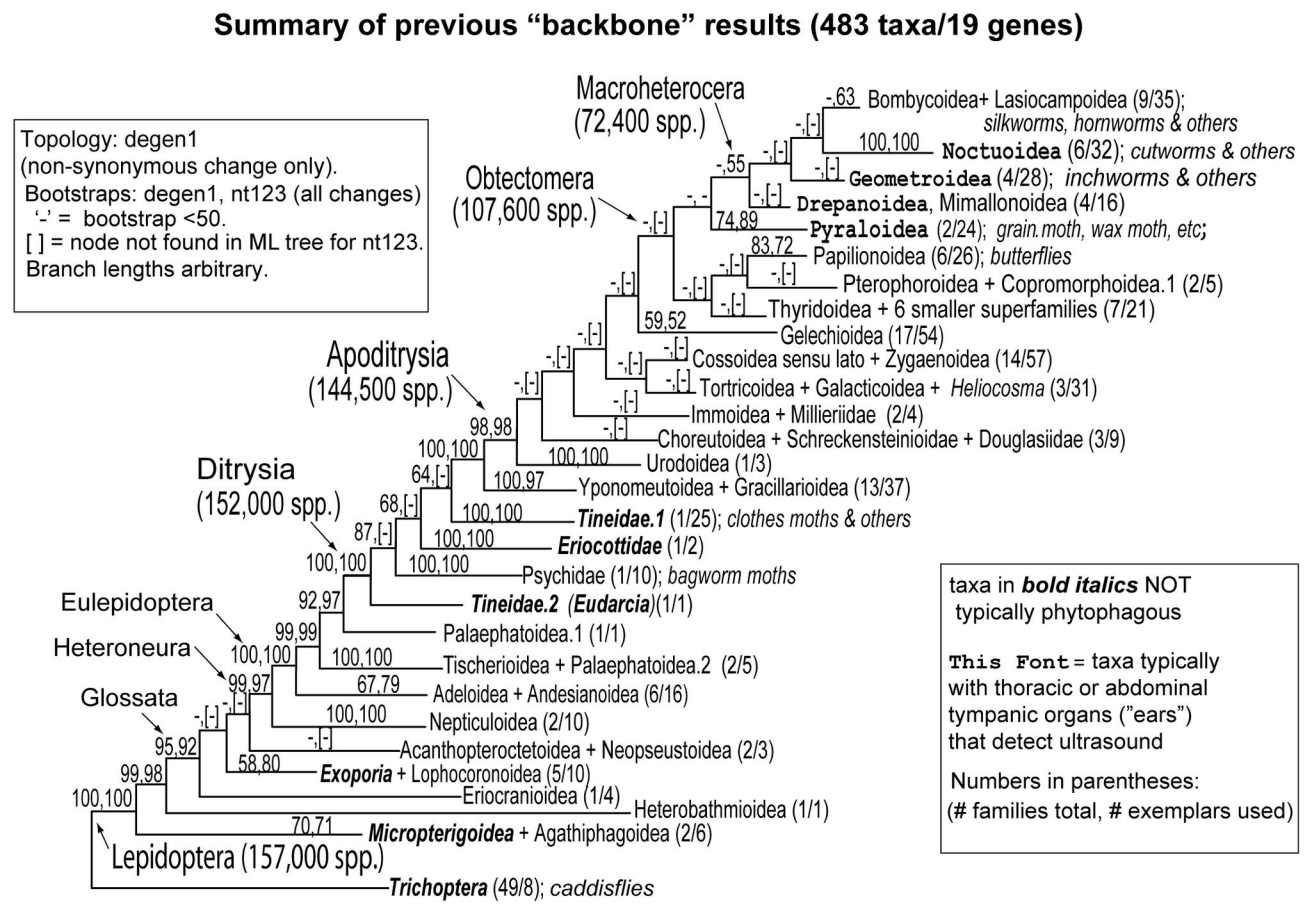
Summary of previous “backbone” phylogeny results (483 taxa/19 genes), modified from Regier et al.[22] ML topology shown for degen1 (non-synonymous change only) is based on 100 GARLI searches. Bootstrap percentages are degen1 followed by nt123 (all nucleotides), based on 1000 bootstrap replicates with 15 search replicates each. Only values greater than 50% are shown. Branch lengths are arbitrary. ‘-’ = node not found in ML tree for nt123. Numbers in parentheses after taxon names indicate number of families/number of exemplars studied. Names in bold denote clades in which larvae are not typically phytophagous. Names in serif font denote clades in which adults typically bear ultrasound-detecting tympanic organs on the thorax and/or abdomen. Classification follows van Nieukerken et al. [1].

The past few years have likewise seen the first attempts at “backbone” phylogenies spanning much or all of the order [19-21]. A recent such study [22], with the largest gene and taxon sampling to date, used 483 exemplars, representing 115 of the approximately 125 families of Lepidoptera [23], sequenced for up to 19 nuclear protein-encoding genes/14.7 kb. It gave a topology quite similar to those of earlier nuclear gene studies, but with stronger bootstrap support. It also agrees with newly-emerging evidence from whole mitochondrial genomes (e.g., [24,25]; see Discussion). The main conclusions of the Regier et al. study [22] are summarized in Figure 1.

The so-called non-ditrysian lineages (Figure 1, left side) are mostly species-poor but rich in morphological variation, and often have apparently relictual distributions suggesting great age. Exhaustive comparative-anatomical studies of these groups (e.g., 26-28), an early application of Hennigian phylogenetics, yielded many synapomorphies and a well‑resolved backbone phylogeny. Although important puzzles remain, the molecular data strongly resolve a majority of these early divergences, recovering previously-recognized major clades including Glossata, Heteroneura and Eulepidoptera (Figure 1). There is also strong molecular support for several novel proposals, such as apparent non-monophyly of Palaephatidae. The molecular data strongly corroborate the clade Ditrysia, named for the presence in the female Terminalia of separate openings for mating and for oviposition, which contains over 98% of lepidopteran species and 80% of the families. 

The superfamilies of Ditrysia, in contrast to the non-ditrysians, tend to be species-rich, cosmopolitan and less distinct morphologically, so that major groupings have been difficult to discern. The authoritative morphological hypothesis synthesized by Kristensen and collaborators [23,29,30] postulated only 11 tentative monophyletic groupings among the 33 ditrysian superfamilies recognized. Molecular data markedly strengthen resolution for the initial divergences within Ditrysia. There is now strong molecular support (Figure 1) for the morphological inference that all Ditrysia apart from Tineoidea form a monophyletic group. Molecular data also strongly support four new or previously uncertain conclusions: (1) The Tineoidea themselves are paraphyletic with respect to all other Ditrysia; (2) Yponomeutoidea and Gracillarioidea are sister groups; (3) Yponomeutoidea and Gracillarioidea together form the sister group to the remaining Ditrysia; and (4), the remaining ditrysians form a strongly supported group consisting of Apoditrysia in an earlier sense [31,32] plus Gelechioidea. Apoditrysia sensu novo [1], now including Gelechioidea, are also supported by several morphological synapomorphies [8,33].

In striking contrast to those in earlier-originating clades, “backbone” relationships in the Apoditrysia sensu lato are almost entirely lacking in strong support from either molecules or morphology, although rogue taxon removal [34] helps somewhat. Recent large-scale molecular studies consistently recover monophyly of some variant of the huge group Obtectomera (107,551 spp.; [1]), originally proposed for families with relatively immobile pupae [31], but support is very weak (Figure 1). Molecular studies also find the large superfamily Gelechioidea to be closely related to Obtectomera, but again with weak support (Figure 1). Within Obtectomera, the morphological working hypothesis recognized a group Macrolepidoptera, consisting of the butterflies (Papilionoidea; 18,363 spp. [1]) and the familiar large moths (inchworms, cutworms, silkmoths and relatives; 5 superfamilies, 72,398 spp.; [1]). Molecular studies have instead consistently separated the butterflies from the large moths, and found that the latter, termed the Macroheterocera [1], are more closely related to the non-macrolepidopteran superfamily Pyraloidea (15,587 spp.; [1]). These findings too, however, have weak bootstrap support (Figure 1). Within Macroheterocera, neither nuclear genes nor morphology provide strong evidence for any relationships at all among superfamilies (Figure 1; but see 24,25). This phylogenetic uncertainty, in turn, limits the power of analyses of the origins, ages and evolutionary consequences of traits hypothesized to promote the spectacular diversification of Apoditrysia, which include 144,524 species in 93 families and 26 superfamilies according to a recent classification [1]. 

Low support along the apoditrysian backbone probably reflects rapid diversification, as in other major insect radiations [35,36]. The alternative explanation, of pervasive strong conflict among gene trees, found little support in our earlier studies [19]. If short branches resulting from rapid radiation are the problem, it may be feasible to strengthen resolution by radically increasing the gene sample. Empirical tests of this proposition, however, have been few. In this study we assess the potential of massive gene sampling for resolving the apoditrysian radiation by analyzing 741 gene sequences, obtained through RNA-Seq, in 46 exemplars spanning nearly all major lineages of Apoditrysia. The resulting dramatic but non-uniform increase in bootstrap support illustrates both the power and the complexity of the phylogenomic approach. 

## Materials and Methods

### Taxon sampling and taxon set design

The goal of this study was to assess the degree to which RNA-Seq transcriptome data can increase the support for relationships among the superfamilies of Apoditrysia over that found in our previous 19-gene study [22]. Our 46 exemplars include 42 apoditrysians spanning 16 of 26 superfamilies and 34 of 93 families of Apoditrysia in a recent classification [1]. The distribution of our exemplars across that classification is shown in Table 1, while the collecting locality, accession number and other details for each specimen are given in Table S1. The only large apoditrysian superfamily (>1,000 species) not sampled was Papilionoidea. The phylogenetic position of Papilionoidea is the focus of a forthcoming independent RNA-Seq study that is yielding results similar to those we report below (A. Y. Kawahara, in litt.)

**Table 1:** Classification of exemplar species included, following van Nieukerken et al. **[1]**.

**LEPIDOPTERA** (43 superfamilies, including all those below)
**Hepialoidea**: Hepialidae: *Phymatopus californicus*
**Palaephatoidea**: Palaephatidae: *Palaephatus luteolus*
**DITRYSIA** (29 superfamilies, including all those below)
**Tineoidea**: **Psychidae**: *Thyridopteryx ephemeraeformis*
**Yponomeutoidea**: Yponomeutidae: Yponomeutinae: *Yponomeuta multipunctella*
**APODITRYSIA** (26 superfamilies, including all those below)
**Urodoidea**: Urodidae: *Urodus decens*
**Zygaenoidea**: Epipyropidae: *Epipomponia nawai*
Lacturidae: *Lactura subfervens*
Limacodidae: Limacodinae: *Euclea delphinii*
Megalopygidae: Megalopyginae: *Megalopyge* crispata
Zygaenidae: Zygaeninae: *Zygaena fausta*
**Cossoidea**: Cossidae: Cossinae: *Culama* sp. 5*, Prionoxystus robiniae*; Hypoptinae: *Givira mucidus*; Zeuzerinae: *Psychogena personalis*; Cossulinae: *Spinulata maruga*
Dudgeoneidae: *Archaeoses polygrapha*
Sesiidae: Sesiinae: *Podosesia syringae*, *Vitacea polistiformis*
**Tortricoidea**: Tortricidae: Olethreutinae: Grapholitini: *Cydia pomonella*; Olethreutini: *Phaecasiophora niveiguttana*
**Immoidea**: Immidae: *Imma tetrascia*
**Choreutoidea**: Choreutidae: Choreutinae: *Hemerophila diva*
**Pterophoroidea**: Pterophoridae: Pterophorinae: *Emmelina monodactyla*
**Gelechioidea**: Amphisbatidae: *Psilocorsis reflexella*
Elachistidae: *Antaeotricha schlaegeri*
Gelechiidae: *Dichomeris punctidiscella*
**OBTECTOMERA** (12 superfamilies, including all those below)
**Thyridoidea**: Thyrididae: Striglininae: *Striglina suzukii*
**Pyraloidea**: Crambidae: Crambinae: *Catoptria oregonica*
Pyralidae: Galleriinae: *Galleria melonella*
**Mimallonoidea**: Mimallonidae: *Lacosoma chiridota*
**MACROHETEROCERA** (5 superfamilies)
**Lasiocampoidea**: Lasiocampidae: Macromphaliinae: *Tolype notialis*
**Bombycoidea**: Bombycidae: Bombycinae: *Bombyx mori*
**Drepanoidea**: Drepanidae: Cyclidiinae: *Cyclidia substigmaria*; Thyatirinae: *Pseudothyatira cymatophoroides*
Cimeliidae: *Axia margarita* (formerly in its own superfamily; Kristensen, 2003)
Doidae: *Doa* sp. (formerly in Noctuoidea; Kristensen, 2003)
**Geometroidea**: Epicopeiidae: *Epicopeia hainesii* (formerly in Drepanoidea; Kristensen, 2003)
Uraniidae: Epipleminae: *Calledapteryx dryopterata*
Geometridae: Ennominae: *Biston betularia*; Geometrinae: *Chlorosea margaretaria*; Sterrhinae: *Idaea* sp. 5
**Noctuoidea**: Erebidae: Lymantriinae: *Lymantria dispar*; Noctuidae: Heliothinae: *Helicoverpa zea*, *Heliothis virescens*; Noctuinae: *Striacosta albicosta*

See Table S1 for accession number, collecting locality and life stage used.

As outgroups we used two non-apoditrysian Ditrysia and two non-ditrysians. For two taxa we used previously published data: for *Bombyx mori*, we used the published genome (SilkDB; [37]), and for *Striacosta albicosta*, we reassembled raw sequences from an earlier study that used older sequencing technology [38]. The purpose of including *S. albicosta* was to gauge how much data can be extracted from such older transcriptome studies, and whether these data can be successfully incorporated into a phylogeny estimate based mainly on newer, larger transcriptome assemblies. For the other 44 taxa we generated transcriptomes *de novo* by RNA-Seq. We matched the taxa included as closely as possible to those in our previous backbone study [22]. Thirty-eight of the 44 species had been included in that study, and for a majority of these we were able to use the same specimen. Four other species were congeners of taxa in the earlier study, and an additional two belonged to the same subfamily and tribe (see Table S1). These substitutions were made because no more material of the same species or genus, respectively, was available. All of the specimens we sequenced came from the ATOLep collection built by the Assembling the Lepidoptera Tree of Life project (Leptree), and had been stored in 100% ethanol at -80° C, some for more than 20 years. 

Taxon sampling in this exploratory study expanded in phases, from 16 to 38 to 46 exemplars, each with a separate phylogenetic analysis, as we sought to characterize the data and develop our informatic and analytical workflows. The initial test set focused (14/16 taxa) on one especially problematic tree region, the hypothesized group consisting of Cossoidea + Sesioidea + Zygaenoidea [30,32]. This assemblage, here termed the “CSZ clade”, consists of 5996 species in 19 families according to van Nieukerken et al. [1], who merged Sesioidea into Cossoidea. It is one of very few groupings among apoditrysian superfamilies that is postulated in the morphology-based working hypothesis [29]. It also presents an exceptionally clear superfamily-level contrast in a major life history feature, internal versus external feeding: Cossoidea and Sesioidea are mostly stem borers, whereas Zygaenoidea are mostly external folivores. In analyses with the 19 Leptree genes (14.7 kb), the CSZ clade is only sometimes monophyletic, and always with very weak support [22]. A core subset of Zygaenoidea is reliably monophyletic, but Sesioidea, Cossoidea and Cossidae never are. Relationships of the sesioid families, the cossoid families and subfamilies, and the two aberrant (parasitic) families of Zygaenoidea (Epipyropidae and Cyclotornidae), to each other and to the “core” Zygaenoidea, are almost completely unsupported (e.g., Figure 1). The test data set also included one non-apoditrysian outgroup (*Yponomeuta*) and one putative apoditrysian outgroup, *Bombyx mori*.

After testing and improving our protocols using the 16-taxon test set, we added 22 more exemplars representing most of the other major lineages of Apoditrysia, focusing on the other large superfamilies (those with over 2000 species). Another eight taxa were then added for a final, 46-taxon analysis. These eight had been held back from the second analysis because we considered them especially likely to complicate tree estimation, either because they have much less data than the rest (*Striacosta albicosta*) or because they were previously identified as difficult-to-place or “rogue” taxa [22]. We wanted to see how much the inclusion/exclusion of such taxa would affect the results based on our very large gene samples. 

An additional, related benefit to our stepwise increase in sampling is the evidence it provides on the effects of taxon sampling density, which has been of special concern in phylogenomics [39-41]. Strong conflicts among phylogenies of 16 and 38 and 46 taxa could suggest the presence of false signal due to taxon under-sampling, as could strong support in the RNA-Seq phylogenies for nodes contradicting strongly supported nodes in the much larger Leptree taxon sample (Figure 1). Successive expansion of the taxon sample could also identify instances in which weak support is increased by denser taxon sampling.

To provide a controlled assessment of the potential benefits of massively increased gene sampling, we compared topologies and branch supports from RNA-Seq analyses both to those from the 19-gene, 483-taxon “backbone” phylogeny [22], and to new 19-gene analyses of 16-, 38- and 45-taxon data sets. The data sets for the 19-gene analyses were taken from the data matrix of Regier et al. [22]. For each species in the RNA-Seq data set, an associated Leptree exemplar from Regier et al. [22], listed in Table S1, was chosen to match it as closely as possible, and was used in our 19-gene analyses. In 38 cases, exactly the same species was used; a closely related substitute was used in six others. For *Striacosta albicosta*, not included in the “backbone” study, we substituted the con-tribal *Agrotis ipsilon*, included by Regier et al. [22], in the 19-gene analysis. We thought it unnecessary to substitute for *Heliothis virescens*, for which we also lack 19-gene data, because it already had a close relative in the 19-gene data set (*Helicoverpa zea*). Thus, the final 19-gene analysis used 45 exemplars instead of 46. 

### RNA-Seq data generation

Total RNA was extracted using Promega SV total RNA isolation mini-kits. The great majority of our specimens were adults; four were larvae (see Table S1), with species identifications verified by comparison of COI sequences with those in the Barcode of Life Data System [42]. For larger moths we used the thorax and/or anterior part of the abdomen; for a few smaller ones we used the entire body. RNA extracts were submitted to the University of Maryland-Institute for Bioscience and Biotechnology Research Sequencing Core. The quality of total RNA was assessed by capillary electrophoresis on an RNA chip using an Agilent Bioanalyzer 2100 system. RNA preps of sufficient quality were subjected to poly-A selection and indexed library construction for sequencing on an Illumina HiSeq1000. Following Hittinger et al. [43] our libraries were left un-normalized, so as to favor highly-expressed genes likely to be present in most species and life stages. Libraries were run four per lane, yielding about 110 million 100-bp paired-end reads per taxon. 

### Sequence quality control and transcript assembly

Reads that did not pass the default Illumina HiSeq1000 “Chastity” quality filter (~5-20% per sample), and those with Phred quality score [44] not greater than 20 at greater than 90% of positions (~5-15% per sample) were discarded. The filtered reads input to assembly (mean = 76M per sample) had median Phred scores greater than 35 for over 95% of the bases in each read.

*De novo* transcriptome assembly was performed using both Trinity (versions r2012‑03‑17 and r2013-02-25 [45]) and Trans-ABySS (versions 1.3.2 and 1.4.4; ABySS versions 1.3.3 and 1.3.5 [46,47]), and the results compared (see Table 2) for numbers and length of transcripts using standard assembly metrics such as N50 (the length *N* for which 50% of all bases are contained in contigs of length *L* < *N*). A typical Trinity assembly required greater than 100 GB RAM and finished in 24 to 96 hours using 16 computer cores. A typical Trans-ABySS run required less than 4 GB RAM and a single processor, finishing in 1-2 hours. The same is true for each constituent ABySS run, of which there were 23 per sample (*k* ranging from 52 to 96 in steps of two). In general, Trinity used more RAM and more compute time, and produced fewer transcripts, than Trans-ABySS, but it produced longer transcripts (Table S2). Combining the Trinity and Trans-ABySS assemblies proved early on to yield a slightly more complete data matrix than either alone, which is why we continued to use both. The added cost of doing so was minimal once a workflow was established.

**Table 2:** Notable changes in topology and bootstrap support with change in taxon sample size, for 741- gene, consensus analyses.

Contrast**^*1*^**	Node	Bootstrap value		
		16 taxa	38 taxa	46 taxa
1	Noctuoidea + Drepanidae	NA**^*2*^**	54	[-]**^*3*^**
1a	Noctuoidea + Geometroidea + Bombycoidea + Lasiocampoidea	NA	[-]	100
1b	Drepanidae + Doidae + Cimeliidae	NA	NA	100
2	Cossoidea + Sesioidea + core Zygaenoidea (CSZ clade)	83	[-]	[-]
2b	Cossoidea + core Zygaenoidea + Obtectomera	[-]	43	57
3	CSZ clade + Obtectomera	NA	90	21

*1* 1a and 1b, and 2b, are alternative groupings that conflict with nodes 1 and 2, respectively.

*2* ‘NA’ = not applicable; node not present because the constituent exemplars are not included in that data set.

*3* [-] = node not present in either ML tree or bootstrap majority rule consensus tree for that data set

Some modification of these methods was necessary for reassembly of the *Striacosta albicosta* transcriptome [37]. We acquired the original 75-bp single-end Illumina reads, which were based on 16 individuals and normalized cDNA, and were not subjected to a “Chastity” filter. Application of our Phred filter eliminated 61% of the reads. We modified Trans-ABySS to work with single-end data, and optimized its k-mer sweep for 75-bp reads (*k* ranged from 38 to 74 in steps of two). The original assembly contained 16,850 contigs of median length 173 bp; our combined Trinity and Trans-ABySS assembly yielded 336,829 contigs of median length 114 bp, including over 15,000 contigs of median length 351 bp from the Trinity assembly alone.

### Orthology determination

To infer orthology, we used HaMStR (version 9; [48]), which in turn uses BLASTP [49], GeneWise [50], and HMMER [51], to search the combined assembly data for protein sequences matching a set of “known” orthologs. The “known” orthologs in our case consisted of a database of 1579 profile hidden Markov models (pHMMs; [52]) of orthologous sequence groups called the “Insecta Hmmer3-2 core-ortholog set”, obtained from the HaMStR web site. These models are based on six genomes representing three holometabolous insect orders (Hymenoptera: *Apis*; Coleoptera: *Tribolium*; Lepidoptera: *Bombyx*); a non-insect pancrustacean (Vericrustacea: *Daphnia*); a different arthropod subphylum (Chelicerata: *Ixodes*); and a different phylum (Annelida: *Capitella*). An annotated list of the putative orthologs in the Insecta Hmmer3-2 data set can be found at http://www.deep-phylogeny.org/hamstr/download/datasets/hmmer3/.

In the first step of the HaMStR procedure, regions of our transcript assemblies (expressed as amino acid sequences) that matched any one of the 1579 Insecta core-ortholog pHMMs were provisionally assigned to the corresponding orthologous group. To reduce the number of highly divergent, potentially paralogous sequences returned by this initial search, we changed the E-value cutoff defining a “hit” to 10^-5^, from the HaMStR default of 1.0, and retained only the top-scoring quartile of hits. In the next HaMStR step, the provisional “hits” from the Insecta search were compared to a “reference taxon” (*Bombyx mori*), and retained only if they survived a reciprocal best BLAST hit test with that taxon. Once assigned to orthologous groups, protein sequences from our assemblies were aligned using MAFFT [53]. The resulting protein alignments were then converted to the correct corresponding nucleotide alignments, using a custom Perl script that substitutes for each amino acid the proper codon from the original coding sequence.

Following initial orthology assignments, we computed “coverage per base” for each orthologous group, defined as read length x median number of reads mapped to orthologous group sequences ÷ median length of orthologous group sequences. Read mappings used Bowtie (version 0.12.8; [54]), allowing up to four mismatches.

### Data matrix construction and paralogy filtering

Our orthology determination pipeline often yields multiple sequences for a particular taxon-locus combination, which can reflect the presence of, among other possibilities, multiple orthologs, heterozygosity, alternatively spliced transcripts, paralogy (including inparalogs; [55]), and sequencing errors. One general approach for reducing this variation to a single sequence, as required for phylogenetic analysis, is exemplified by the “representative” option in HaMStR [48]. This procedure chooses the single sequence (or concatenation of non-overlapping fragments) with the best pairwise alignment to a chosen reference taxon. We developed an alternative that accommodates the uncertainty in orthology determination by combining the set of sequences into a single consensus sequence, using nucleotide ambiguity codes [56] as necessary. Consensus sequences were generated by providing the alignment of the nucleotide coding sequences corresponding to the amino acid sequences passing our filtering steps, described above, to the consensus_iupac BioPerl subroutine [57]. There are two principal motivations for this “consensus” approach. The first is a desire to incorporate all information about specific nucleotide states for positions that might reasonably be inferred to be orthologous, including those where orthologous relationships among genes between pairs of taxa are many to one, and many to many, as well as cases of polymorphism. A second motivation is to mitigate the effects of mistaken orthology determination and other errors, including those resulting from incorrect choice of a single representative sequence, by in effect reducing the weight of positions at which transcription fragments differ. By including more available transcription fragments, moreover, consensus can potentially yield longer total sequences than representative, as has been our experience. However, degenerating nucleotide sites that vary among transcripts could result in dilution of phylogenetic information, if the single best sequence chosen by representative were almost always the most phylogenetically appropriate one. The approach that works best is thus an empirical question, which we addressed by performing both procedures and comparing the results. 

Despite the filters described above, inspection of our initial 1579 alignments revealed obvious paralogs. An extreme example is orthologous group 412460 of the Insecta Hmmer3-2 database, annotated there as acetyl-CoA acetyltransferase, a type of thiolase. In our data, HaMStR search returned two divergent sets of sequences for this ortholog group, which upon BLAST search matched two different members of the thiolase gene family in a noctuid moth. No single E-value threshold can eliminate problems of this kind, so we turned to direct scrutiny of gene trees (e.g. [58-60]). Using the initial 16 test taxa, a maximum likelihood (ML) gene tree was constructed for each orthologous group using all matching sequences, and provided as input to the program PhyloTreePruner [61]. If the sequences for a particular taxon form a polyphyletic group, the program prunes the gene tree to the maximal subtree in which the non-polyphyly criterion is met for all taxa. For the 16 test taxa, PhyloTreePruner pruned 838 of the 1579 gene trees to some degree. For this exploratory study we have taken a very conservative action based on these results, using for all subsequent phylogenetic analyses only the 741 genes in which no evidence of paralogy was found in the test taxa, and entirely ignoring the remaining genes; alternative possibilities for future studies are considered in the Discussion section. Following application of the paralogy filter, the 741 putative ortholog alignments were concatenated, adding gaps for missing data as necessary using a custom Perl script. For all phylogenetic analyses the nucleotide matrix was subjected to degen1 coding (version 1.4; [62]), and sites not represented by sequence data in at least four taxa were subsequently removed. “Degen” uses degeneration coding to eliminate all synonymous differences among species from the data set, resulting in phylogeny inference based only on non-synonymous nucleotide change. This procedure was shown in our previous backbone study [22] to generally improve recovery of deep nodes. At deeper levels in the Lepidoptera, inclusion of synonymous change in any form, even as part of a codon model, sometimes introduces conflict and systematic error, due to compositional heterogeneity [21,22]. Analysis under degen1 can be viewed as a computationally efficient approximation to a purely “mechanistic” amino acid model, i.e., one based on the genetic code but not incorporating empirical transition frequencies between amino acids [21,63,64].

 Sequences and alignments for the 19-gene analyses were extracted from Table S4 of the Leptree backbone study [22]. Nine of these genes are present in the Insecta Hmmer3-2 database. The PCR amplicon codes of these nine from Regier et al. [22] are: 40fin, 109fin, 192fin, 262fin, 265fin, 268fin, 3007fin, 3070fin, and CAD. Five of these genes were eliminated by our paralogy screen, while the following four, listed by their numbers in the Insecta Hmmer3-2 database, were included among the 741 used in phylogenetic analyses: 413101 - 262fin; 412564 - 268fin; 412293 - 265fin; 412031 - 40fin.

### Phylogenetic analysis

Maximum likelihood phylogenetic analyses used GARLI (Genetic Algorithm for Rapid Likelihood Inference; version 2.0 [65]) and grid computing [66,67] via a web service on molecularevolution.org [68] based on tools developed by Bazinet et al. [69] that include post-processing with DendroPy [70], R [71], and custom Perl scripts. The majority of the phylogenetic analyses were completed using the BOINC volunteer computing platform [72] (http://boinc.umiacs.umd.edu). We used a GTR+I+G nucleotide model together with GARLI default settings, including random stepwise addition starting trees, except that we halved the number of successive generations yielding no improvement in likelihood score that prompts termination (genthreshfortopoterm = 10000), as suggested for bootstrapping in the GARLI manual. Memory requirements ranged from 800 MB for the 16-taxon, 741-gene analysis to 3500 MB for the 46-taxon, 741-gene analysis. Each best tree was selected from 100 GARLI search replicates, while bootstrap analyses consisted of 1000 replicates. Insufficient search effort during bootstrapping has been shown to artificially depress bootstrap support (BP) values [22]. A rough guide to the effort needed is provided by our initial 100 ML search: if the best tree topology is found only rarely, multiple search replicates per bootstrap replicate may be necessary. We tested each of our data sets for the effect of increased search effort on BP values, at levels of one, five, and ten search replicates per bootstrap replicate. We found a significant increase in BP values for several analyses using five search replicates instead of one, but did not find a significant improvement using ten search replicates instead of five. Thus, all results presented here used five search replicates per bootstrap replicate.

The 741-gene and 19-gene data matrices have been deposited in Dryad (doi:10.5061/dryad.02qv3). The Illumina reads have been deposited in the NIH Sequence Read Archive, as BioProject PRJNA222254.

## Results

### Data matrix properties

The paralogy-filtered matrix of 741 genes contained from 742,017 to 873,036 nucleotide positions and was 80-93% complete, depending on the number of taxa included and the orthology determination procedure used (Table S3). Thus, overall matrix completeness was slightly higher than in the 14.7 kb, 483-taxon Leptree analysis [22]. Completeness was fairly consistent among the 44 newly-sequenced taxa, ranging from, e.g., 67% to 84% for the 46-taxon, 741-gene consensus matrix (Table S2). Our reassembly of the previously-published *Striacosta albicosta* sequence reads [38] yielded sequence for 1138 orthologous groups, whose median sequence length was 147 bp. Thus, in the paralogy-filtered 46-taxon data matrices, for example, *Striacosta* has approximately half the data of our other taxa (Table S2). Coverage per base (Table S4) averaged 103× for 15 test taxa, with a range of 31× to 334×.

### Phylogenetic results

**Figure 2:**
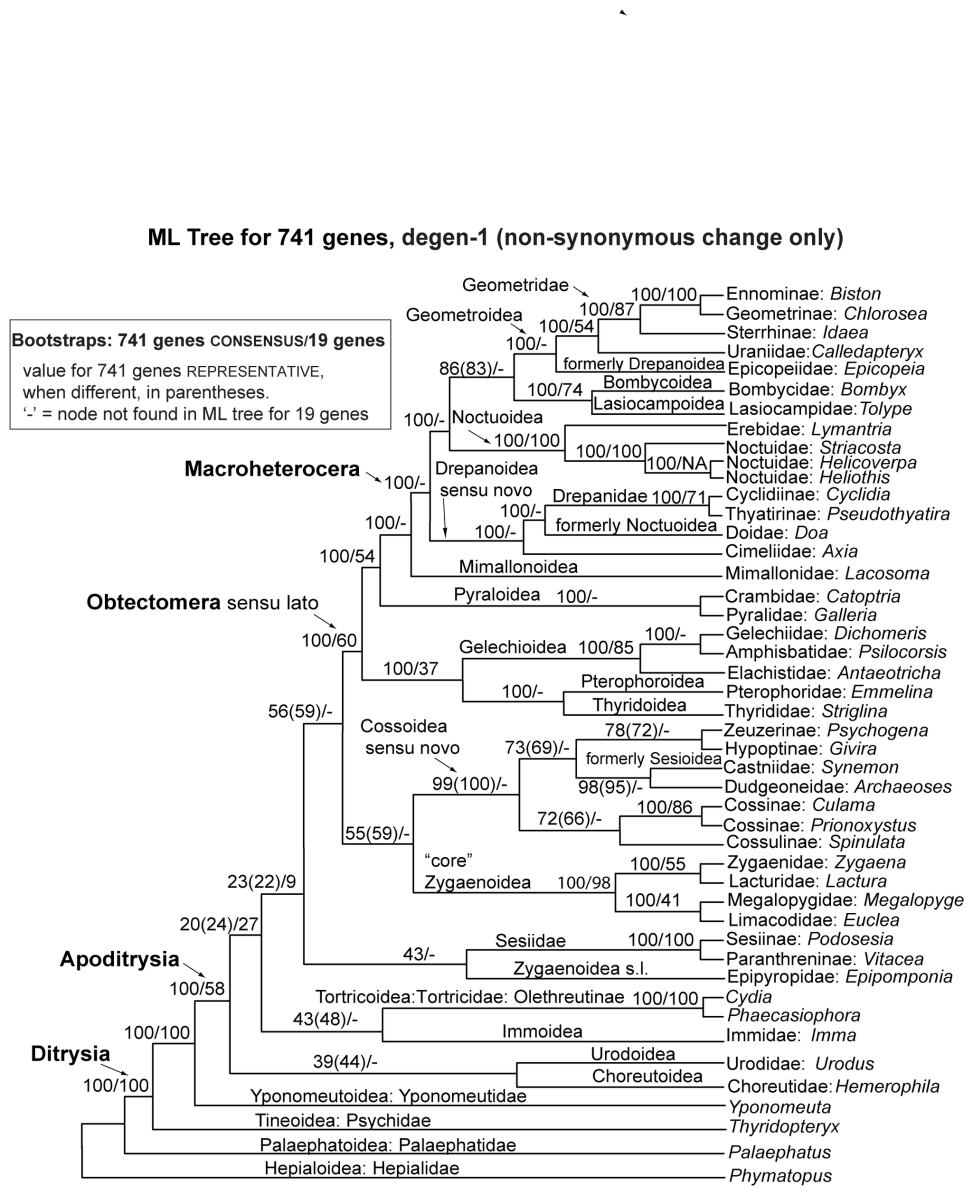
ML tree for 46 taxa, 741 paralogy-filtered genes, degen-1 (non-synonymous change only). Bootstrap percentages: 741 genes consensus method, followed by 741 genes Rrepresentative method in parentheses but only when these two differ, followed by 19 genes, each based on 1000 bootstrap replicates with 5 search replicates each. ‘-’ = node not found in ML tree for 19 genes.

 The tree of maximum likelihood found for both the 46-taxon, 741-gene consensus data set and its representative counterpart is shown in Figure 2, together with bootstrap values for the consensus and representative 46-taxon, 741-gene data sets and the 45-taxon, 19-gene data set. A phylogram version of the same tree is given in Figure S1. ML cladograms and bootstrap values for all other data sets are given in Figures S2–S5.

The two alternative procedures for determining a single sequence per taxon-locus combination for phylogenetic inference when orthology search returns multiple “hits”, i.e., representative and consensus, yielded identical ML topologies, and nearly identical bootstrap values (Figure 2). A marked difference between the two procedures was observed in the 38-taxon analysis, for which finding the best tree topology took considerably more search effort for representative than for consensus: out of 100 ML searches, the best tree topology was found 25 times for the consensus matrix, but only once for the representative matrix. However, we found no such difference for either the 16- or 46-taxon analyses; in those cases, a comparable amount of search effort for each procedure was required to find the best tree topology. An experiment described in Supplementary Text S1suggested that the greater search effort required for representative in the 38-taxon case stems from conflicting signal in a small proportion of nucleotide positions in that matrix which are left ambiguous in the consensus matrix.

The most dramatic pattern in the results is the much greater frequency, across all taxon sets, of strong support for nodes subtending multiple superfamilies in the 741-gene analyses than in either the corresponding 19-gene analyses or the 483-taxon “backbone” study. For example, in the 46-taxon, 741-gene ML topology of Figure 2, there are 22 nodes within Apoditrysia that subtend taxa assigned to different superfamilies in either the newest classification [1] or its immediate predecessor [23]. Of these, 11 have bootstrap support (BP) of 100%, two additional nodes have BP ≥98%, and one additional node has BP >80%, for a total of 14/22 nodes with “strong” or “very strong” support (Figure 2). In contrast, of 23 nodes subtending multiple superfamilies in the ML topology for the 45-taxon, 19-gene matrix (Figure S1), none have BP ≥80%; only one has BP >70%, and only three have BP >50% (Figures 2, S1).

Strong deeper-node support in the 741-gene analyses is not spread evenly across the Apoditrysia, but is restricted almost entirely to a clade consisting of Obectomera sensu van Nieukerken et al. [1] + Gelechioidea + Pterophoroidea (Figure 2). Of the 12 nodes within and including that clade which subtend multiple subfamilies in recent classifications, 11 have BP = 100% and all have BP >80%. In contrast, of the 11 such nodes elsewhere among the Apoditrysia, none have BP=100% and only two have BP >80%.

Tree topology changed little as taxon sampling expanded for 741 genes. Table 2 summarizes the main differences in topology and bootstrap support among the 16-, 38- and 46-taxon analyses. In no comparison among trees for different numbers of taxa were there incompatible nodes that each had strong bootstrap support. Thus, there is little evidence for artifactual strong support resulting from taxon undersampling. The most notable conflict concerns monophyly of the putative CSZ clade. In the 16-taxon analysis, which includes only one apoditrysian (*Bombyx*) apart from the putative CSZ clade, that clade gets 82% bootstrap support (Figure S5). In contrast, the 38- and 46-taxon analyses, which include many other apoditrysian lineages, find the CSZ assemblage to be paraphyletic with respect to the clade Obtectomera + Gelechioidea + Pterophoroidea. Bootstrap support for this conclusion, however, is only 43% and 59% for 38 and 46 taxa, respectively (Figures 2, S3). The most striking instance of decline in bootstrap without change in topology involves the grouping of the “CSZ clade” constituents with the Obtectomera, to the exclusion of other apoditrysians. The 90% bootstrap support for this grouping in the 38-taxon analysis falls to 27% in the 46-taxon analysis, which includes three additional non-obtectomeran superfamilies.

The evidence is stronger for a positive effect of taxon sampling density on node support. The clearest examples are the contrasting positions of Noctuoidea and Drepanidae in the 38- versus 46-taxon, 741-gene analyses. In the 38-taxon analysis (Figure S3), which is missing several small groups (Cimeliidae, Axiidae, Doidae) that may or may not represent distinct superfamilies of Macroheterocera [1,23], Noctuoidea are grouped with Drepanidae, but with weak support (BP = 54%). When the three missing groups are added, as part of the 46-taxon analysis, Drepanidae and Noctuoidea are no longer paired, but the new positions of these two taxa, together with those of the newly-added families, are all supported by BP=100%. A beneficial effect of denser taxon sampling on node support is also suggested by the generally lower support in our new 19-gene analyses of 16, 38 and 46 taxa than in our previous 19-gene, 483-taxon study [22]. For example, bootstrap support for Apoditrysia, 98% in Regier et al. [22], is only 58% here in the 19-gene, 45-taxon analysis. Moreover, unlike the 483-taxon study, the 19-gene, 45 taxon analysis also fails to support monophyly for Pyraloidea and for Macroheterocera. An interaction between gene and taxon sampling is suggested, finally, by the fact that the 45-taxon, 741-gene analysis supports the monophyly of both Pyraloidea and Macroheterocera with BP=100%. 

## Discussion

Our results suggest that the expansive gene sampling yielded by RNA-Seq may be able to strongly resolve inter-superfamily relationships throughout a clade consisting of Obtectomera sensu van Nieukerken et al. [1] plus Gelechioidea and Pterophoroidea (at least), comprising over two-thirds of the species of Lepidoptera. But, might these high bootstraps be misleading? Multiple authors have urged caution in the interpretation of bootstrap support in phylogenomic studies (e.g., [40,73]) or even abandonment of bootstraps altogether in favor of other support measures [74]. If random error is sufficiently reduced by massive gene sampling, strong but misleading bootstrap support might arise from even subtle forms of pervasive systematic error, such as minor compositional heterogeneity or slight differences in the relative abundance of strongly-conflicting gene tree topologies, as well as from long-branch attraction due to the typically sparse taxon sampling in phylogenomics.

How could we judge whether the strong support seen in our results is artifactual? That explanation would gain credence if the strongly supported nodes repeatedly conflicted with groupings that were robustly supported, or at least consistently monophyletic, in previous studies. In fact, however, the topology of the RNA-Seq phylogeny of Figure 2 is closely similar, though not identical, to that of the 483-taxon, 19-gene study (Figure 1) and to those of earlier molecular studies [19-21]. It is also consistent, in topology and node support levels, with recent studies using whole mitochondrial genomes [24,25]. All strongly-supported relevant nodes from previous nuclear gene studies are also strongly supported by the RNA‑Seq analysis. Nowhere in the tree does a strongly supported node in the phylogenomic study contradict a strongly supported node in any earlier study. Moreover, it appears that limited taxon sampling, rather than inducing artifacts, can be better overcome by the RNA-Seq data than by the 19-gene data: in the 38- and 46-taxon analyses, the RNA-Seq data strongly support the monophyly of Pyraloidea, for which previous molecular and morphological evidence is definitive, whereas the 19-gene data fail to group the two pyraloid exemplars.

A second reasonable expectation, if strong support in the phylogenomic results were largely artifactual, is that such support should be distributed across all levels in the tree. Indeed, some of the forces that can produce strong false signal, such as convergence in amino acid composition and long branch attraction, should be more likely for deeper than for shallower divergences. But in fact, within Apoditrysia, strong support from RNA-Seq is concentrated in the subordinate clade Obtectomera, while the deeper divergences have uniformly weak support. 

These observations – agreement of strong support with previous groupings, and decreasing signal strength with increasing depth of divergence within Apoditrysia – suggest that such strong support as we find in the RNA-Seq results is real rather than artifactual. They further suggest that even with 741 genes, we are still data-limited: we do not yet have enough characters to fully resolve all stages of the rapid radiation of the Apoditrysia. On the plus side, however, it also appears that, unlike many previous phylogenomic studies, we are not working with levels of divergence at which strong bootstrap support, even from entirely non-synonymous change, is both inevitable and often misleading [40,73,74].

If, as we argue, the strong support seen in our 741-gene analyses is real, it appears that further taxon sampling could quickly produce major advances in our understanding of the huge clade Obtectomera. Precise definition of this clade has been difficult, and the placement of multiple superfamilies has been unclear. Our results suggest that there is a sharp discontinuity between superfamilies that are and are not strongly supported as near relatives of the Macroheteroceran moths. If this distinction holds up under further taxon sampling, it would be reasonable to use it to define the Obtectomera, which would then include both Gelechioidea and Pterophoroidea. It appears that RNA-Seq may be able to definitively resolve all or nearly all relationships within Obtectomera so redefined. There is very strong support for monophyly of Macroheterocera sensu van Nieukerken et al., and for Mimallonidae as the sister group to these. It might make sense to include Mimallonidae in Macroheterocera. There is also very strong support for a sister group relationship of Mimallonidae + Macroheterocera to Pyraloidea. 

All of the superfamilies of Macroheterocera are sampled here, and relationships among them, with one possible exception, are all strongly supported. The basal divergence is between a clade consisting of Cimeliidae + (Doidae + Drepanidae) and one containing the remaining four superfamilies; an identical or similar division, albeit weakly supported, is seen in previous molecular studies. The first grouping corroborates the recent incorporation of all three families into Drepanoidea sensu novo [1], and increases the evidence for removal of *Doa* from Noctuoidea, despite its possession of the two main noctuoid morphological synapomorphies. Within the clade consisting of Noctuoidea, Geometroidea, Bombycoidea and Lasiocampoidea, the latter two are strongly grouped, and only the node uniting these with Geometroidea (BP=84%) has bootstrap support of less than 100%. The position of Epicopeiidae, weakly supported in all previous studies, strongly corroborates their transfer from Drepanoidea to Geometroidea [1,22]. The close relationship between Geometroidea and Bombycoidea + Lasiocampidae suggested here may explain why Epicopeiidae sometimes grouped (weakly) with the latter in earlier studies [20]. 

Although Papilionoidea, formerly grouped with the “big moths”, were not included in this study, one can confidently predict, from earlier studies (Figure 1), that they would fall among the “lower” Obtectomera. In Figure 2 this would mean, somewhere between the base of Obtectomera and the base of Pyraloidea + Macroheterocera. This prediction has recently been strongly confirmed by studies based on mitochondrial genomes [24,25] and on RNA-Seq (A. Y. Kawahara, in litt.), although the exact sister group of the butterflies will not be known until sampling of the non-macroheteroceran superfamilies of Obtectomera is complete.

While prospects for resolving the Obtectomera sensu lato look promising, the outlook is less bright in the “lower,” i.e. non-obtectomeran, Apoditrysia. In this tree region only two nodes subtending multiple current or former superfamilies get bootstrap support approaching conclusive levels (Figure 2). There is 99% bootstrap support for a clade consisting of Cossoidea sensu stricto [23] plus Castniidae, formerly placed in Sesioidea [23,38]. If this grouping holds up under further RNA-Seq sampling, it may be useful to redefine Cossoidea to conform to it. Such a definition would re-exclude Sesiidae, included here by van Nieukerken et al., [1], for which no strong placement has been discovered. Within the putative Cossoidea sensu novo, only a single inter-family relationship gets notable bootstrap support, namely, the novel pairing of Castniidae with Dudgeonidae (BP=98%). The relationships of the four cossid subfamilies sampled, to each other and to Castniidae + Dudgeonidae, have weaker support (BP = 71-81%). Elsewhere in the non-obtectomeran Apoditrysia, no bootstrap value exceeds 59%. Phylogenetic relationships in the Cossoidea-Zygaenoidea-Sesioidea complex will clearly need much further work.

Why are the “lower” Apoditrysia such a difficult phylogenetic problem, in comparison to lepidopteran lineages of both greater and lesser age? Several complementary explanations seem plausible. Cladogenesis might have been particularly rapid at the base of Apoditrysia as compared to later on, resulting in especially short internal branches. Alternatively, the rate of subsequent extinction might have been high, reducing the taxon sample available for reconstructing rapid cladogenesis. Or, these divergences might be harder to reconstruct simply because they are older than those in Obtectomera, leaving more time for synapomorphies to be overwritten by subsequent substitution. Increasing the gene sample might allow us to overcome the first and third effects. To overcome the second effect, we would want to sample taxa as densely as possible, but would face limits set by extinction. Fortunately, as our results so far have shown, gene and taxon sampling are to some degree interchangeable. Therefore, more gene sampling might help in this case as well. Thus, further expanding the gene sample may be critical to further resolution of the lower Apoditrysia, no matter why these lineages are so refractory to phylogenetics. 

One immediate way to increase our gene sample would be to relax our severe initial interpretation of the PhyloTreePruner results, under which only genes for which no evidence of paralogy was found were considered suitable for phylogenetic analysis. Following Kocot et al. [61], one could recover some of the information thereby lost by estimating bootstrap support for the individual gene trees and avoiding pruning when support is weak. One could also include the partially incomplete pruned gene trees, from which the apparent paralogs have been deleted, in phylogeny calculations. While these measures might be useful, a potentially more profitable approach in the long run would be to address the underlying problem that led us to PhyloTreePruner in the first place. The Insecta Hmmer3-2 database was a highly useful starting point, but for two reasons it is not ideal for studies within Lepidoptera. First, it contains only the 1579 genes that were identifiably orthologous across six very divergent arthropod and annelid genomes. Comparisons restricted to Lepidoptera would undoubtedly yield a much higher number of useful genes; for example, the complete proteome of the diamondback moth (Yponomeutoidea: Plutellidae: *Plutella xylostella*) is close to 15,000 genes [75]. Second, presumably because most of the taxa on which the database is built are so divergent from Lepidoptera, many of its putative ortholog groups appear to include sequences that are non-orthologous in Lepidoptera. Therefore, it would be useful to have a new database of Lepidoptera-specific gene models for orthology determination in the Apoditrysia. Such an effort could capitalize on a growing set of annotated lepidopteran genomes and transcriptomes, which now includes multiple apoditrysians as well as a member of the sister group to Apoditrysia [75-78]. 

## Summary and Conclusions

This study explored the potential of next-generation sequencing to conclusively resolve relationships among the superfamilies of advanced ditrysian Lepidoptera (Apoditrysia), which were very weakly supported in previous nuclear gene studies. We used RNA-Seq to generate 1579 putatively orthologous gene sequences across a taxonomically broad sample of 40 apoditrysians plus four outgroups, to which we added two taxa using previously published data. Phylogenetic analysis of a 46-taxon, 741-gene matrix, resulting from a strict filter that eliminated ortholog groups containing any apparent paralogs, yielded dramatic overall increase in bootstrap support for deeper nodes within Apoditrysia as compared to results from previous and concurrent 19-gene analyses. High support was restricted mainly to the huge apoditrysian subclade Obtectomera broadly defined, in which 11 of 12 nodes subtending multiple superfamilies had bootstrap support of 100%. The strongly supported nodes showed little conflict with groupings from previous studies, and were little affected by changes in taxon sampling, suggesting that they reflect true signal rather than artifacts of massive gene sampling. Additional taxon sampling has the potential to definitively resolve obtectomeran superfamily relationships. In contrast, strong support was seen at only 2 of 11 deeper nodes among the “lower”, non-obtectomeran apoditrysians. These represent a much harder phylogenetic problem, for which further increase in gene sampling, together with improved orthology assignments, offers one potential path to resolution. 

## Supporting Information

Figure S1 **ML phylogram and bootstraps for the 46-taxon, 741-gene, consensus analysis.** The topology and consensus bootstraps are identical to those in Figure 2.
(TIF)

Figure S2 **ML cladogram and bootstraps for the 45-taxon, 19-gene analysis.**
(TIF)

Figure S3 **ML cladogram and bootstraps for the 38-taxon, 741-gene, consensus analysis.**
(TIF)

Figure S4 **ML cladogram and bootstraps for the 38-taxon, 19-gene analysis.**
(TIF)

Figure S5 **ML cladogram and bootstraps for the 16-taxon analyses.** (A) the 16-taxon, 741-gene consensus analysis, (B) the16-taxon, 741-gene representative analysis (C) the 16-taxon, 19-gene analysis.
(TIF)

Table S1 **Exemplars used for RNA-Seq and their distribution across the classification of van Nieukerken et al. [1], accession numbers, life stages and collection localities.**
(XLSX)

Table S2 **Summary statistics for RNA-Seq reads and assemblies.**
(XLSX)

Table S3 **Size and completeness of aligned data matrices from RNA-Seq.**
(XLS)

Table S4 **RNA-Seq coverage for 15 test taxa.**
(XLSX)

Text S1 **Exploration of differences between consensus and representative methods for determining a single sequence per taxon-locus combination for phylogenetic inference.**
(DOCX)

## References

[1] van Nieukerken EJ, Kaila L, Kitching IJ, Kristensen NP, Lees DC, et al. (2011) Order Lepidoptera Linnaeus, 1758. In Zhang Z.-Q. Animal Biodiversity: An outline of higher level classification and survey of taxonomic richness. Order Lepidoptera Linnaeus, 1758. Zootaxa 3148: 212-221

[2] Wagner DL (2001) Moths. In Levin SA Encyclopedia of Biodiversity. Academic Press, San Diego, CA. pp. 249-270.

[3] Roe AD, Weller SJ, Baixeras J, Brown J, Cummings MP, et al. (2009) Evolutionary framework for lepidoptera model systems. In Goldsmith M.R. Marec F. Molecular Biology and Genetics of the Lepidoptera. CRC Press / Taylor & Francis (Contemporary topics in entomology series). Boca Raton, Florida. pp. 1-24

[4] Ehrlich PR, Raven PH (1964) Butterflies and plants: A study in coevolution. Evolution 18: 586–608. doi:10.2307/2406212.

[5] Mitter C, Farrell B, Wiegmann BM (1988) The phylogenetic study of adaptive radiation: Has phytophagy promoted insect diversification? Am Nat 132: 107-128. doi:10.1086/284840.

[6] Winkler IS, Mitter C (2008) The phylogenetic dimension of insect/plant interactions: a review of recent evidence In: Tillmon K, Specialization, Speciation, and Radiation: The Evolutionary Biology of Herbivorous Insects. University of California Press pp. 240-263.

[7] Zwick A, Regier JC, Mitter C, Cummings MP (2010) Increased gene sampling yields robust support for higher-level clades within Bombycoidea (Lepidoptera). Syst Entomol 33: 190-209.

[8] Kaila L, Mutanen M, Nyman T (2011) Phylogeny of the mega-diverse Gelechioidea (Lepidoptera): adaptations and determinants of success. Mol Phylogenet Evol 61: 801-809. doi:10.1016/j.ympev.2011.08.016. PubMed: 21903172. 21903172

[9] Young CJ (2006) Molecular relationships of the Australian Ennominae (Lepidoptera: Geometridae) and implications for the phylogeny of the Geometridae from molecular and morphological data. Zootaxa 1264:1-147.

[10] Yamamoto S, Sota T (2007) Phylogeny of the Geometridae and the evolution of winter moths inferred from a simultaneous analysis of mitochondrial and nuclear genes. Mol Phylogenet Evol 44: 711–723. doi:10.1016/j.ympev.2006.12.027. PubMed: 17363285. 17363285

[11] Sihvonen P, Mutanen M, Kaila L, Brehm G, Hausmann A et al. (2011) Comprehensive molecular sampling yields a robust phylogeny for geometrid moths (Lepidoptera: Geometridae). PLOS ONE 6: e20356. doi:10.1371/journal.pone.0020356. PubMed: 21673814. 21673814 PMC3106010

[12] Kawahara AY, Ohshima I, Kawakita A, Regier JC, Mitter C et al. (2011) Increased gene sampling provides stronger support for higher-level groups within gracillariid leaf mining moths and relatives (Lepidoptera: Gracillariidae). BMC Evol Biol 11: 182. doi:10.1186/1471-2148-11-182. PubMed: 21702958. 21702958 PMC3145599

[13] Mitchell A, Mitter C, Regier JC (2006) Systematics and evolution of the cutworm moths (Lepidoptera: Noctuidae): evidence from two protein-coding nuclear genes. Syst Entomol 31: 21-46.

[14] Zahiri R, Kitching IJ, Lafontaine JD, Mutanen M, Kaila L et al. (2011) A new molecular phylogeny offers hope for a stable family level classification of the Noctuoidea (Lepidoptera). Zool Scr 40: 158-173.

[15] Heikkilä M, Kaila L, Mutanen M, Peña C, Wahlberg N (2012) Cretaceous origin and repeated tertiary diversification of the redefined butterflies. Proc R Soc of London B 279: 1093-1099. PubMed: 21920981. 10.1098/rspb.2011.1430 PMC3267136 21920981

[16] Regier JC, Mitter C, Solis MA, Hayden JE, Landry B et al. (2012) A molecular phylogeny for the pyraloid moths (Lepidoptera: Pyraloidea) and its implications for higher-level classification. Syst Entomol 37: 635–656. doi:10.1111/j.1365-3113.2012.00641.x.

[17] Regier JC, Brown JW, Mitter C, Baixeras J, Cho S et al. (2012) A molecular phylogeny for the leaf-roller moths (Lepidoptera: Tortricidae) and its implications for classification and life history evolution. PLOS ONE 7: e35574. doi:10.1371/journal.pone.0035574. PubMed: 22536410. 22536410 PMC3334928

[18] Sohn J-C, Regier JC, Mitter C, Davis D, Landry J-F et al. (2013) A molecular phylogeny for Yponomeutoidea (Insecta, Lepidoptera, Ditrysia) and its implications for classification, biogeography and the evolution of host plant use. PLOS ONE 8: e55066. doi:10.1371/journal.pone.0055066. PubMed: 23383061. 23383061 PMC3561450

[19] Regier JC, Zwick A, Cummings MP, Kawahara AY, Cho S et al. (2009) Toward reconstructing the evolution of advanced moths and butterflies (Lepidoptera: Ditrysia): an initial molecular study. BMC Evol Biol 9: 280. doi:10.1186/1471-2148-9-280. PubMed: 19954545. 19954545 PMC2796670

[20] Mutanen M, Wahlberg N, Lauri Kaila L (2010) Comprehensive gene and taxon coverage elucidates radiation patterns in moths and butterflies. Proc R Soc of London B 277: 2839-2848. doi:10.1098/rspb.2010.0392. PubMed: 20444718. PMC2981981 20444718

[21] Cho S, Zwick A, Regier JC, Mitter C, Cummings MP et al. (2011) Can deliberately incomplete gene sample augmentation improve a phylogeny estimate for ditrysian Lepidoptera (Hexapoda)? Syst Biol 60: 782-796. doi:10.1093/sysbio/syr079. PubMed: 21840842. 21840842 PMC3193767

[22] Regier JC, Mitter C, Zwick A, Bazinet AL, Cummings MP et al. (2013) A large-scale, higher-level, molecular phylogenetic study of the insect order Lepidoptera (Moths and Butterflies). PLOS ONE 8(3): e58568. doi:10.1371/journal.pone.0058568. PubMed: 23554903. 23554903 PMC3595289

[23] Kristensen NP, editor (2003) Lepidoptera, Moths and Butterflies. Vol. 2: Morphology, Physiology, and Development. In Fischer M Handbook of Zoology 4. Arthropoda: Insecta, part 36. Vol. 2 Vol. 2 Walter de Gruyter, Berlin & New York. 564 pp.

[24] Kim MJ, Kang AR, Jeong HC, Kim KG, Kim I (2011) Reconstructing intraordinal relationships in Lepidoptera using mitochondrial genome data with the description of two newly sequenced lycaenids, *Spindasis* *takanonis* and *Protantigius* *superans* (Lepidoptera: Lycaenidae). Mol Phylogenet Evol 61: 436–445. doi:10.1016/j.ympev.2011.07.013. PubMed: 21816227. 21816227

[25] Lu H-F, Su T-J, Luo A-R, Zhu C-D, Wu C-S (2013) Characterization of the complete mitochondrion genome of diurnal moth *Amata* *emma* (Butler) (Lepidoptera: Erebidae) and its phylogenetic implications. PLOS ONE 8: e72410. doi:10.1371/journal.pone.0072410. PubMed: 24069145. 24069145 PMC3771990

[26] Kristensen NP, Nielsen ES (1983) The *Heterobathmia* life history elucidated: Immature stages contradict assignment to suborder Zeugloptera (Insecta, Lepidoptera). Z Zool Syst Evolut-Forsch 21: 101-124.

[27] Kristensen NP (1984) Studies on the morphology and systematics of primitive Lepidoptera (Insecta). Steenstrupia 10: 141-191.

[28] Davis DR (1986) A new family of monotrysian moths from austral South America (Lepidoptera: Palaephatidae), with a phylogenetic review of the Monotrysia. Smith Contr Zool No. 434.

[29] Kristensen NP (Ed.) (1998) Handbook of Zoology 4. Lepidoptera, moths and butterflies. Walter de Gruyter, Berlin & New York.

[30] Kristensen NP, Skalski AW (1998) Phylogeny and palaeontology. In Kristensen NP Handbook of Zoology 4. Lepidoptera, moths and butterflies. Walter de Gruyter, Berlin & New York pp. 7-25.

[31] Minet J (1986) Ebauche d’une classification modern de l’ordre des Lepidopteres. Alexanor, 14: 291–313.

[32] Minet J (1991) Tentative reconstruction of the ditrysian phylogeny (Lepidoptera: Glossata). Entomol Scand 22: 69–95. doi:10.1163/187631291X00327.

[33] Kaila L (2004) Phylogeny of the superfamily Gelechioidea (Lepidoptera: Ditrysia): an exemplar approach. Cladistics 20: 303–340. doi:10.1111/j.1096-0031.2004.00027.x. 34892939

[34] Aberer AJ, Stamatakis A (2011) A simple and accurate method for rogue taxon identification. IEEE International Conference on Bioinformatics and Biomedicine. Atlanta, Ga.: 118-122.

[35] Whitfield JB, Lockhart PJ (2007) Deciphering ancient rapid radiations. Trends Ecol Evol 22: 258–265. doi:10.1016/j.tree.2007.01.012. PubMed: 17300853. 17300853

[36] Wiegmann BM, Trautwein MD, Winkler IS, Barr NB, Kim JW et al. (2011) Episodic radiations in the fly tree of life. Proc Natl Acad Sci U S A 108: 5690-5695. doi:10.1073/pnas.1012675108. PubMed: 21402926. 21402926 PMC3078341

[37] Duan J, Li R, Cheng D, Fan W, Zha X et al. (2010) SilkDB v2.0: a platform for silkworm (*Bombyx* *mori*). Genome Biology - Nucleic Acids Res 38: D453-D456. doi:10.1093/nar/gkp801. 19793867 PMC2808975

[38] Miller N, Sun J, Sappington T (2012) High-throughput transcriptome sequencing for SNP and gene discovery in a moth. Environ Entomol 41: 997-1007. doi:10.1603/EN11216.

[39] Pick KS, Philippe H, Schreiber F, Erpenbeck D, Jackson DJ et al. (2010) Improved phylogenomic taxon sampling noticeably affects non-bilaterian relationships. Mol Biol Evol 27: 1983–1987. doi:10.1093/molbev/msq089. PubMed: 20378579. 20378579 PMC2922619

[40] Philippe H, Brinkmann H, Lavrov DV, Littlewood DTJ, Manuel M et al. (2011) Resolving difficult phylogenetic questions: why more sequences are not enough. PLoS Biol 9: e1000602. doi:10.1371/journal.pbio.1000602. PubMed: 21423652. 21423652 PMC3057953

[41] Wiens JJ, Tiu J (2012) Highly incomplete taxa can rescue phylogenetic analyses from the negative impacts of limited taxon sampling. PLOS ONE 7: e42925. doi:10.1371/journal.pone.0042925. PubMed: 22900065. 22900065 PMC3416753

[42] Ratnasingham S, Hebert PDN (2007) BOLD: The barcode of life system Available online at: (http://www.barcodinglife.org). Mol Ecol Notes 7: 355-364 10.1111/j.1471-8286.2007.01678.x PMC1890991 18784790

[43] Hittinger CT, Johnston M, Tossberg JT, Rokas A (2010) Leveraging skewed transcript abundance by RNA-Seq to increase the genomic depth of the tree of life. Proc Natl Acad Sci U S A 107: 1476-1481. doi:10.1073/pnas.0910449107. PubMed: 20080632. 20080632 PMC2824393

[44] Ewing B, Green P (1998) Basecalling of automated sequencer traces using phred. II. Error probabilities. Genome Res 8: 186-194. PubMed: 9521922. 9521922

[45] Grabherr MG, Haas BJ, Yassour M, Levin JZ, Thompson DA et al. (2011) Full-length transcriptome assembly from RNA-Seq data without a reference genome. Nat Biotechnol 29: 644-652. doi:10.1038/nbt.1883. PubMed: 21572440. 21572440 PMC3571712

[46] Birol I, Jackman SD, Nielsen CB, Qian JQ, Varhol R et al. (2009) De novo transcriptome assembly with ABySS. Bioinformatics 25: 2872-2877. doi:10.1093/bioinformatics/btp367. PubMed: 19528083. 19528083

[47] Robertson G, Schein J, Chiu R, Corbett R, Field M et al. (2010) De novo assembly and analysis of RNA-seq data. Nat Methods 7: 909-912. doi:10.1038/nmeth.1517. PubMed: 20935650. 20935650

[48] Ebersberger I, Strauss S, von Haeseler A (2009) HaMStR: profile hidden markov model based search for orthologs in ESTs. BMC Evol Biol 9: 157. doi:10.1186/1471-2148-9-157. PubMed: 19586527. 19586527 PMC2723089

[49] Altschul SF, Madden TL, Schäffer AA, Zhang J, Zhang Z et al. (1997) Gapped BLAST and PSI-BLAST: a new generation of protein database search programs. Nucleic Acids Res 25: 3389-3402. doi:10.1093/nar/25.17.3389. PubMed: 9254694. 9254694 PMC146917

[50] Birney E, Clamp M, Durbin R (2004) GeneWise and Genomewise. Genome Res 14: 988-995. doi:10.1101/gr.1865504. PubMed: 15123596. 15123596 PMC479130

[51] Eddy SR (2011) Accelerated profile HMM searches. PLoS Comput Biol 7: e1002195 PubMed: 22039361. 22039361 10.1371/journal.pcbi.1002195 PMC3197634

[52] Eddy SR (1998) Profile hidden Markov models. Bioinformatics 14: 755-763. doi:10.1093/bioinformatics/14.9.755. PubMed: 9918945. 9918945

[53] Katoh K, Frith MC (2012) Adding unaligned sequences into an existing alignment using MAFFT and LAST. Bioinformatics 28: 3144-3146. doi:10.1093/bioinformatics/bts578. PubMed: 23023983. 23023983 PMC3516148

[54] Langmead B, Trapnell C, Pop M, Salzberg SL (2009) Ultrafast and memory-efficient alignment of short DNA sequences to the human genome. Genome Biol 10: R25. doi:10.1186/gb-2009-10-3-r25. PubMed: 19261174. 19261174 PMC2690996

[55] Koonin EV (2005) Orthologs, paralogs, and evolutionary genomics. Annu Rev Genet 39: 309-338. doi:10.1146/annurev.genet.39.073003.114725. PubMed: 16285863. 16285863

[56] Cornish-Bowden A (1985) Nomenclature for incompletely specified bases in nucleic acid sequences: recommendations 1984. Nucleic Acids Res 13: 3021-3030. doi:10.1093/nar/13.9.3021. PubMed: 2582368. 2582368 PMC341218

[57] Stajich JE, Block D, Boulez K, Brenner SE, Chervitz SA et al. (2002) The BioPerl toolkit: Perl modules for the life sciences. Genome Res 12: 1611-1618. doi:10.1101/gr.361602. PubMed: 12368254. 12368254 PMC187536

[58] Dunn CW, Hejnol A, Matus DQ, Pang K, Browne WE et al. (2008) Broad phylogenomic sampling improves resolution of the animal tree of life. Nature 452: 745-749. doi:10.1038/nature06614. PubMed: 18322464. 18322464

[59] Kocot KM, Cannon JT, Todt C, Citarella MR, Kohn AB et al. (2011) Phylogenomics reveals deep molluscan relationships. Nature 477: 452–456. doi:10.1038/nature10382. PubMed: 21892190. 21892190 PMC4024475

[60] Kocot K, Halanych KM, Krug PJ (2013) Phylogenomics supports Panpulmonata: Opisthobranch paraphyly and key evolutionary steps in a major radiation of gastropod molluscs. Mol Phylogenet Evol. doi:10.1016/j.ympev.2013.07.001. 23850501

[61] Kocot KM, Citarella MR, Moroz LR, Halanych KM (2013) Phylotreepruner: a phylogenetic-tree based approach for selection of orthologous sequences for phylogenomics. Available: http://sourceforge.net/projects/phylotreepruner/files/src/. 10.4137/EBO.S12813 PMC3825643 24250218

[62] Zwick A, Regier JC, Zwickl DJ (2012) Resolving discrepancy between nucleotides and amino acids in deep-level arthropod phylogenomics: Differentiating serine codons in 21-amino-acid models. PLOS ONE 7: e47450. doi:10.1371/journal.pone.0047450. PubMed: 23185239. 23185239 PMC3502419

[63] Seo T-K, Kishino H (2009) Statistical comparison of nucleotide-, amino-acid-, and codon-substitution models for the evolutionary analysis of protein-coding sequences. Syst Biol 58: 199–210. doi:10.1093/sysbio/syp015. PubMed: 20525578. 20525578

[64] Yang Z, Nielsen R, Goldman N, Pedersen A-MK (2000) Codon substitution models for heterogeneous selection pressure at amino acid sites. Genetics 155: 431–449. PubMed: 10790415. 10790415 10.1093/genetics/155.1.431 PMC1461088

[65] Zwickl DJ (2006) Genetic algorithm approaches for the phylogenetic analysis of large biological sequence datasets under the maximum likelihood criterion. Ph.D. dissertation, The University of Texas at Austin.

[66] Bazinet AL, Cummings MP (2008) The Lattice Project: a Grid research and production environment combining multiple Grid computing models. In Weber MHW Distributed & Grid Computing — Science Made Transparent for Everyone. Principles, Applications and Supporting Communities. Tectum Publishing, Marburg pp. 2-13.

[67] Cummings MP, Huskamp JC (2005) Grid computing. Educause Review 40: 116-117.

[68] Bazinet AL, Cummings MP (2011) Computing the Tree of Life — Leveraging the power of desktop and service grids (IPDPSW). In 2011 IEEE International Symposium on Parallel and Distributed Processing Workshops and Phd Forum. pp. 1896–1902.

[69] Bazinet AL, Myers DS, Fuetsch J, Cummings MP (2007) Grid services base library: a high-level, procedural application program interface for writing Globus-based Grid services. Future Gener Comp Sy 23: 517-522.

[70] Sukumaran J, Holder MT (2010) DendroPy: A Python library for phylogenetic computing. Bioinformatics 26: 1569-1571. doi:10.1093/bioinformatics/btq228. PubMed: 20421198. 20421198

[71] R Development Core Team (2011) R: A language and environment for statistical computing. R Foundation for Statistical Computing, Vienna, Austria ISBN 3-900051-07-0 Available: http://www.R-project.org/.

[72] Anderson D (2004) BOINC: a system for public-resource computing and storage. Proceedings of the 5th IEEE/ACM International Workshop on Grid Computing, Grid ’04, Washington, DC, USA. pp. 4-10. IEEE Computer Society.

[73] Regier JC, Zwick A (2011) Sources of signal in 62 protein-coding nuclear genes for higher-level phylogenetics of arthropods. PLOS ONE 6: e23408. doi:10.1371/journal.pone.0023408. PubMed: 21829732. 21829732 PMC3150433

[74] Salichos L, Rokas A (2013) Inferring ancient divergences requires genes with strong phylogenetic signals. Nature 497: 327-331. doi:10.1038/nature12130. PubMed: 23657258. 23657258

[75] You M, Yue Z, He W, Yang X, Yang G et al. (2013) A heterozygous moth genome provides insights into herbivory and detoxification. Nat Genet 45: 220-225. doi:10.1038/ng.2524. PubMed: 23313953. 23313953

[76] Dasmahapatra KK, Walters JR, Briscoe AD, Davey JW, Whibley A et al. (2012) Butterfly genome reveals promiscuous exchange of mimicry adaptations among species. Nature 487: 94–98. PubMed: 22722851. 22722851 10.1038/nature11041 PMC3398145

[77] Li Y, Wang G, Tian J, Liu H, Yang H et al. (2012) Transcriptome analysis of the silkworm (*Bombyx* *mori*) by high-throughput RNA sequencing. PLOS ONE 7(8): e43713. doi:10.1371/journal.pone.0043713. PubMed: 22928022. 22928022 PMC3426547

[78] Zhan S, Merlin C, Boore JL, Reppert SM (2011) The monarch butterfly genome yields insights into long-distance migration. Cell 147: 1171-1185. doi:10.1016/j.cell.2011.09.052. PubMed: 22118469. 22118469 PMC3225893

