# Supplementary figures and images for: Can RNA-Seq Resolve the Rapid Radiation of Advanced Moths and Butterflies (Hexapoda: Lepidoptera: Apoditrysia)? An Exploratory Study
Source: PLoS One. 2013 Dec 4;8(12):e82615. doi: 10.1371/journal.pone.0082615 (PMC3853519; doi:10.1371/journal.pone.0082615)

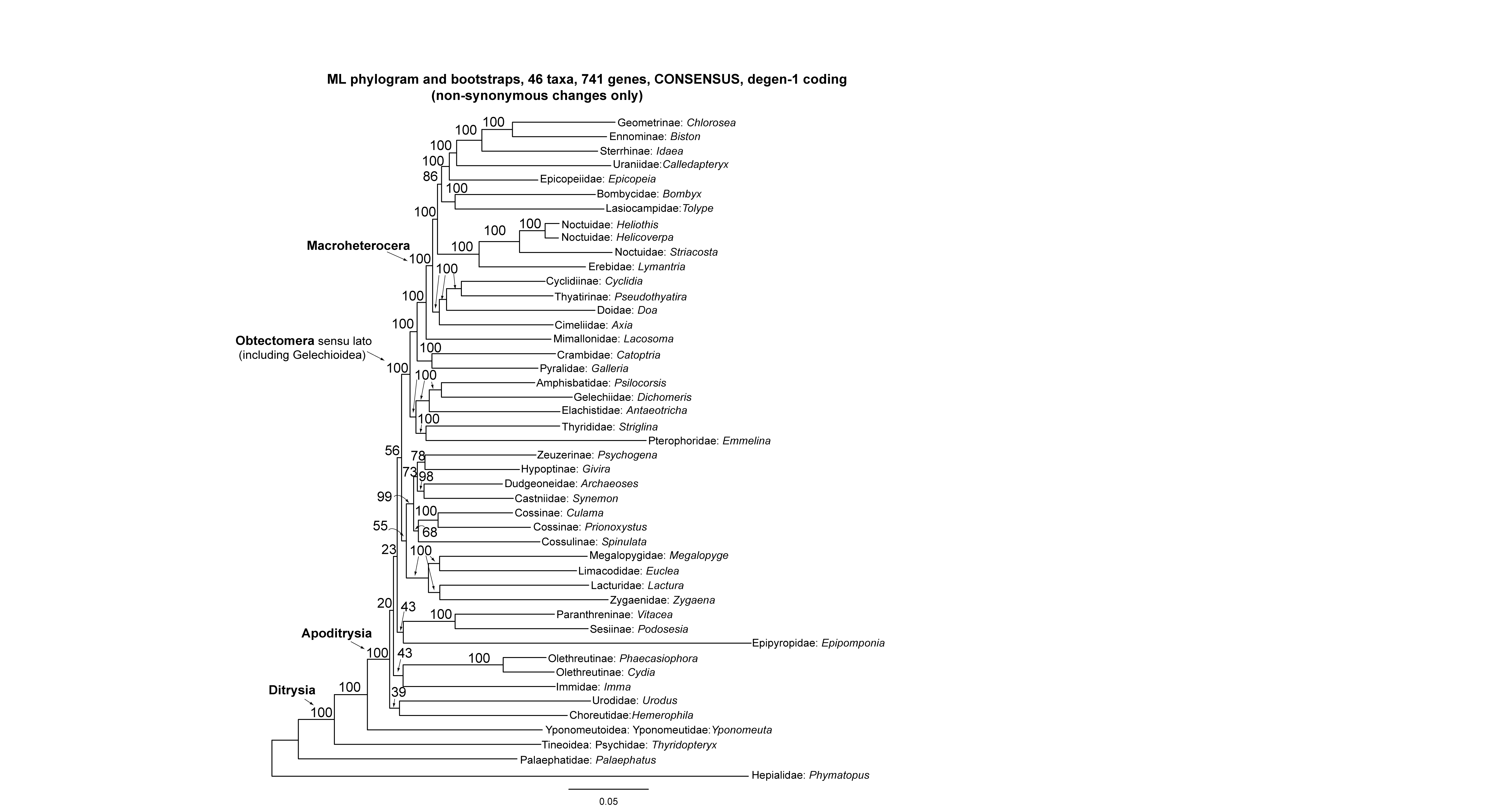

Supplement: Figure S1 — ML phylogram and bootstraps for the 46-taxon, 741-gene, consensus analysis. The topology and consensus bootstraps are identical to those in Figure 2. (TIF) [file pone.0082615.s001.tif]

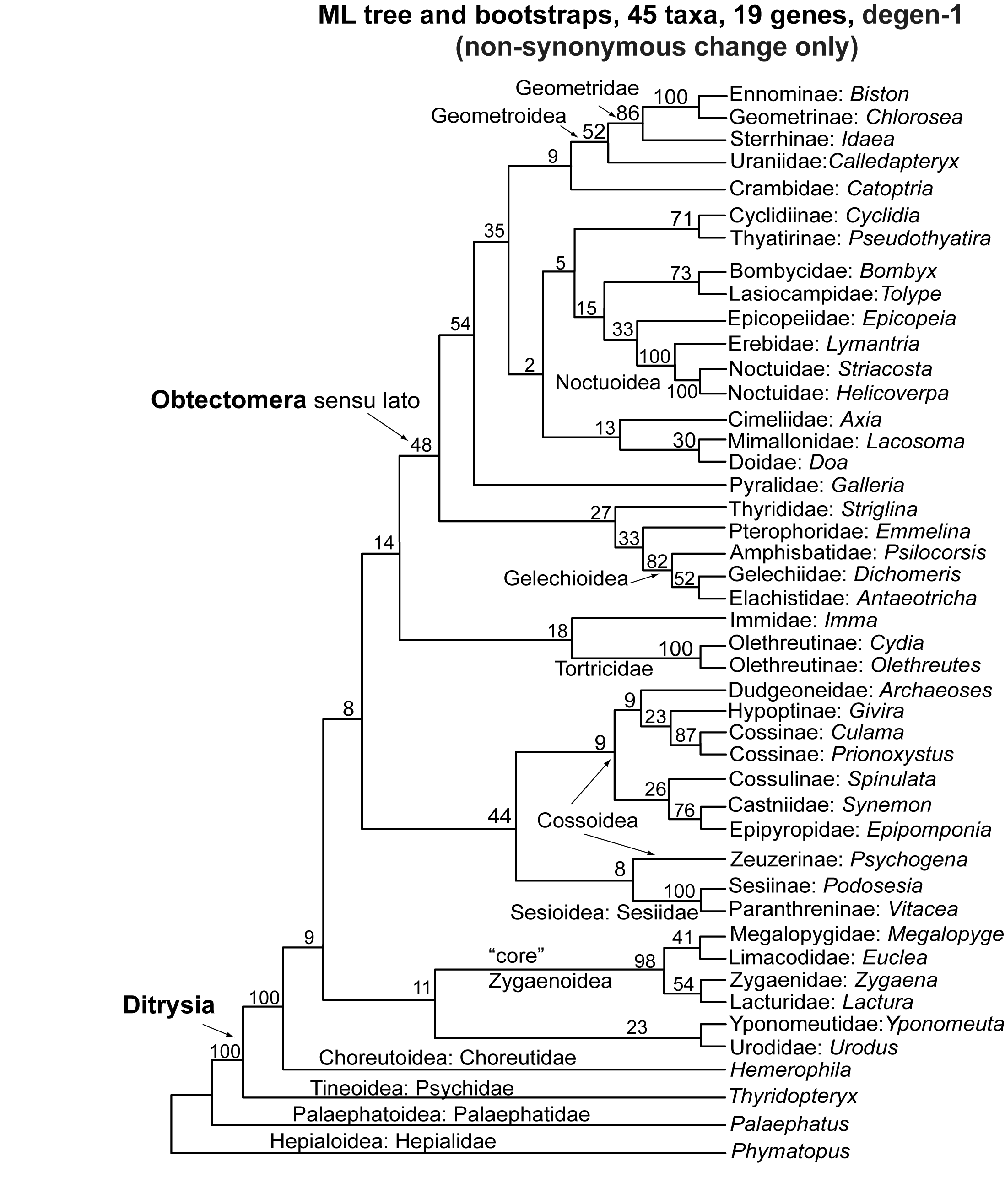

Supplement: Figure S2 — ML cladogram and bootstraps for the 45-taxon, 19-gene analysis. (TIF) [file pone.0082615.s002.tif]

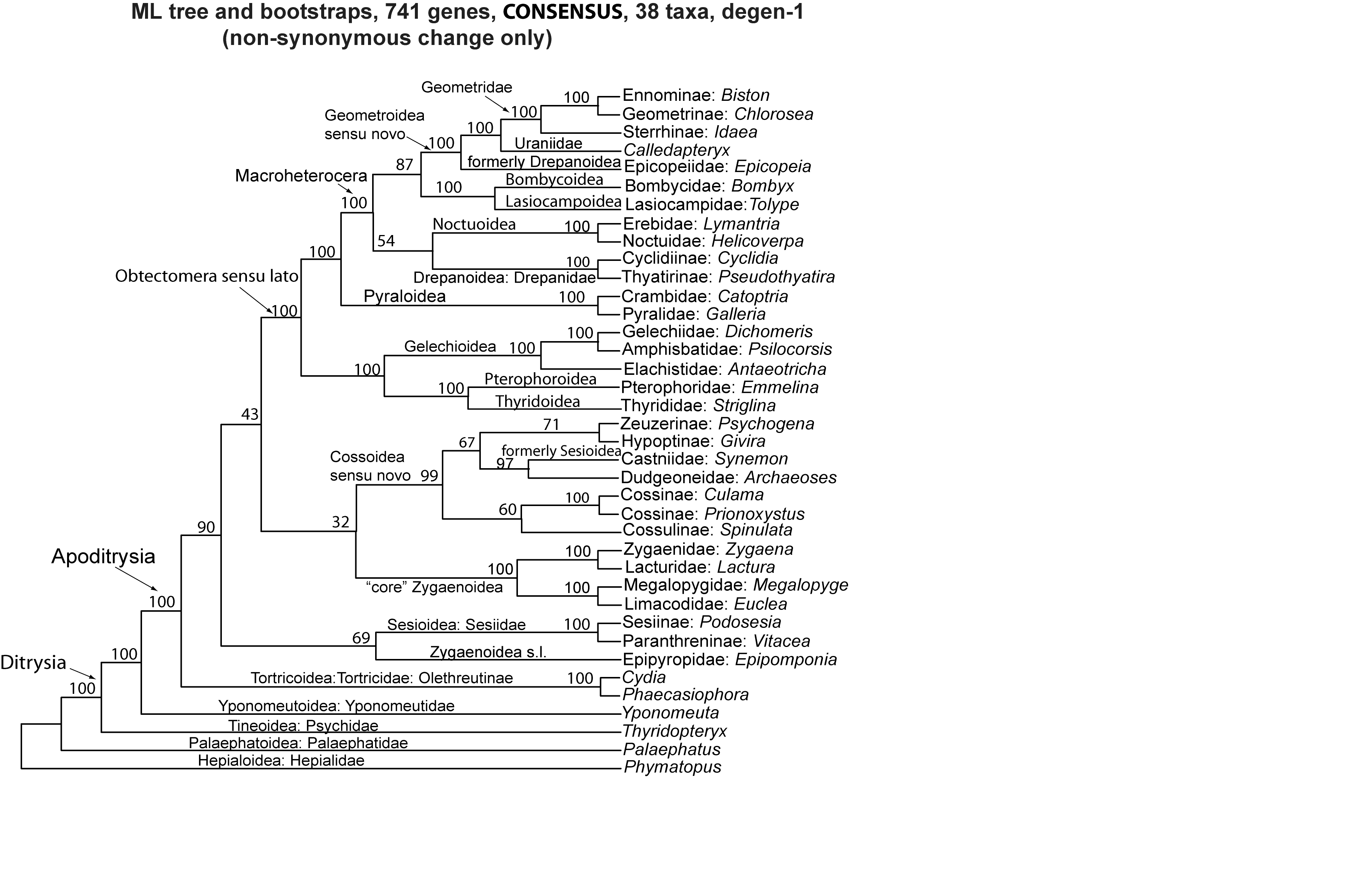

Supplement: Figure S3 — ML cladogram and bootstraps for the 38-taxon, 741-gene, consensus analysis. (TIF) [file pone.0082615.s003.tif]

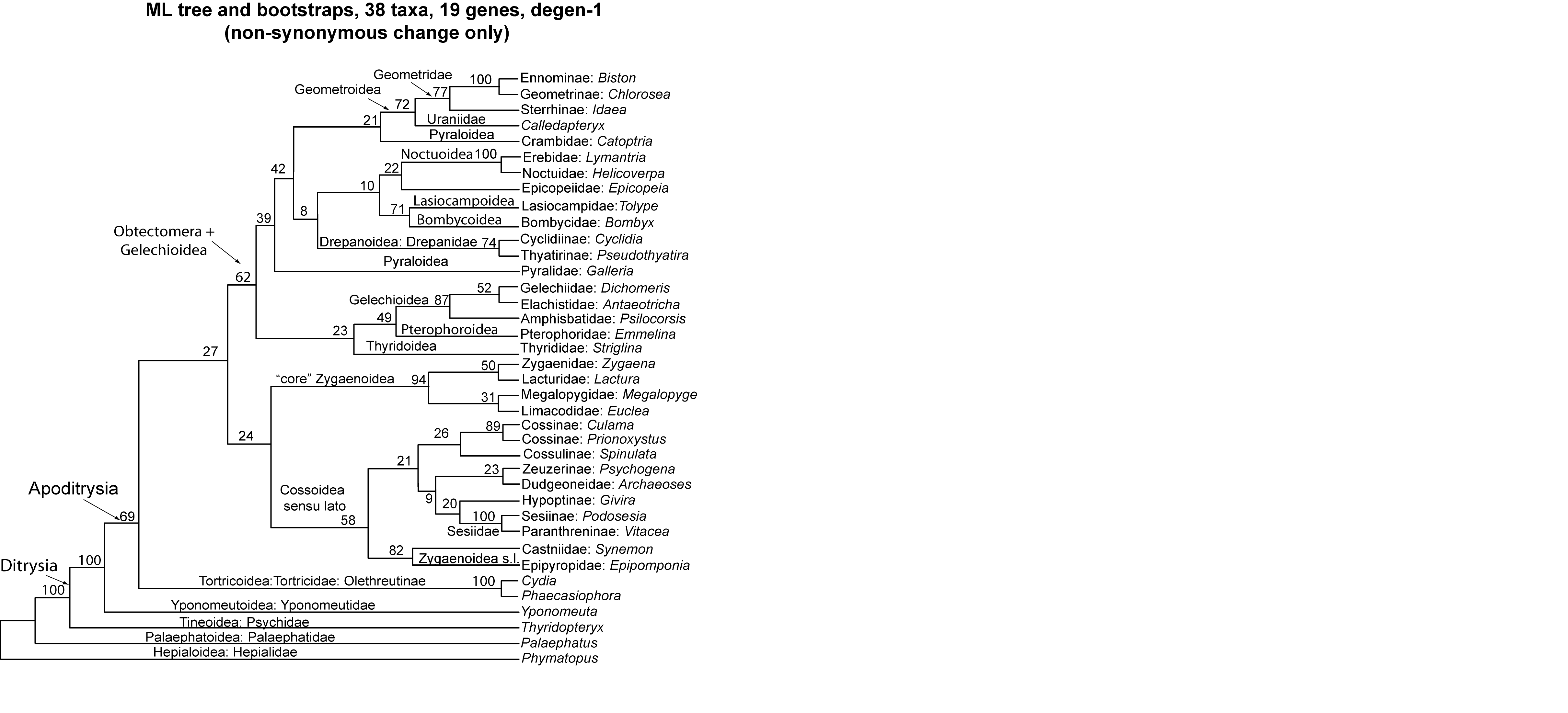

Supplement: Figure S4 — ML cladogram and bootstraps for the 38-taxon, 19-gene analysis. (TIF) [file pone.0082615.s004.tif]

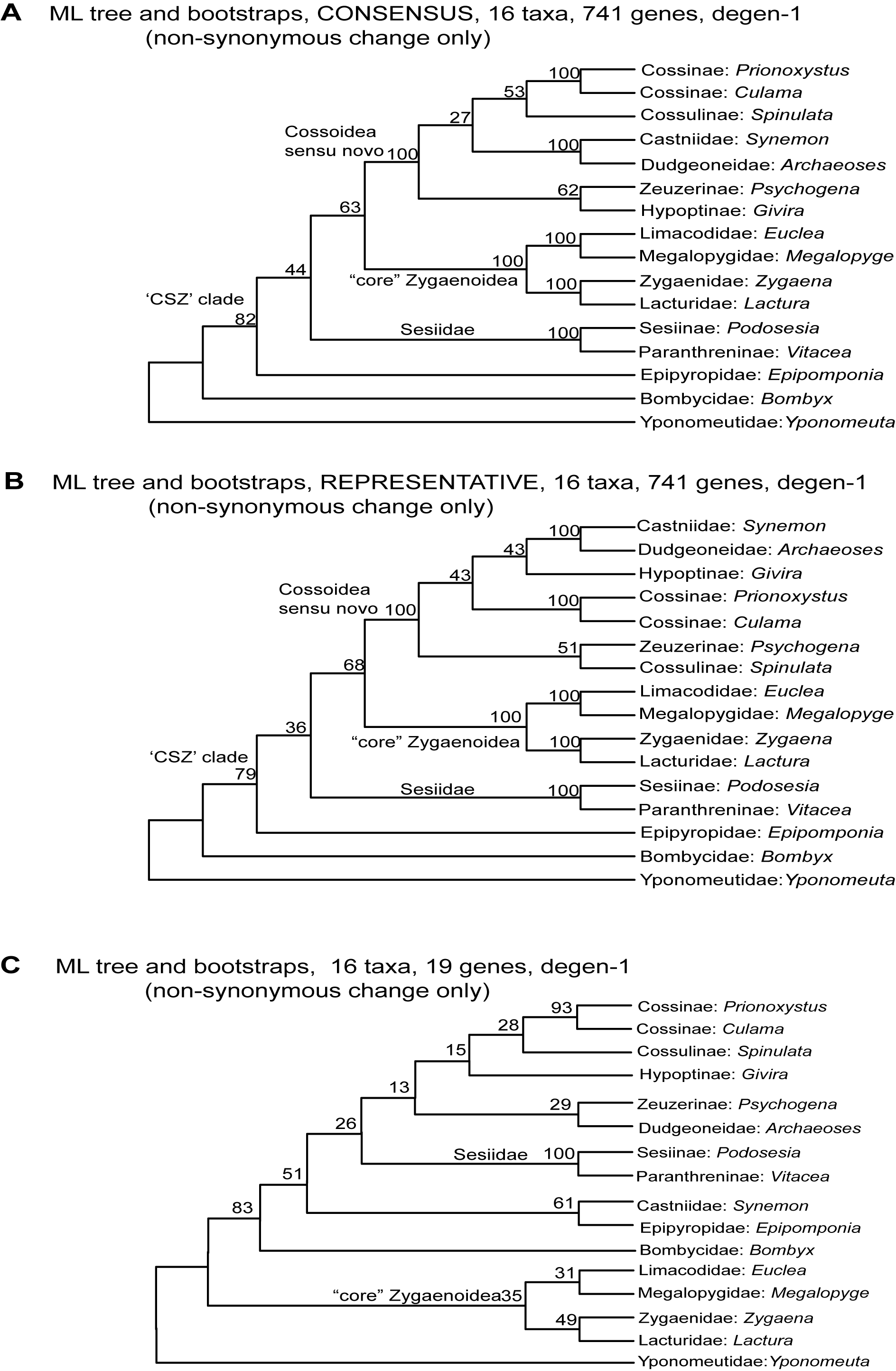

Supplement: Figure S5 — ML cladogram and bootstraps for the 16-taxon analyses. (A) the 16-taxon, 741-gene consensus analysis, (B) the16-taxon, 741-gene representative analysis (C) the 16-taxon, 19-gene analysis. (TIF) [file pone.0082615.s005.tif]
