# Supplementary material for: Can RNA-Seq Resolve the Rapid Radiation of Advanced Moths and Butterflies (Hexapoda: Lepidoptera: Apoditrysia)? An Exploratory Study
Source: PLoS One. 2013 Dec 4;8(12):e82615. doi: 10.1371/journal.pone.0082615 (PMC3853519; doi:10.1371/journal.pone.0082615)
Supplement: Text S1 — Exploration of differences between consensus and representative methods for determining a single sequence per taxon-locus combination for phylogenetic inference. (DOCX) [file pone.0082615.s010.docx]

**Text S1. Exploration of differences between CONSENSUS and REPRESENTATIVE options.**

The two alternative procedures we used for producing a single sequence per taxon‑locus combination for phylogenetic inference when orthology search returns multiple “hits” – i.e., representative and consensus – yielded identical ML topologies, and nearly identical bootstrap values in all analyses (Fig. 2). A marked difference between the two procedures was observed in the 38‑taxon analysis, however, for which finding the best tree topology took considerably more search effort for representative than for consensus: out of 100 ML searches, the best tree topology was found 25 times for the consensus matrix, but only once for the representative matrix. (We found no such difference for either the 16‑ or 46‑taxon analyses.) This appendix describes our efforts to find the cause of the different behaviors of the

representative and consensus options in the 38-taxon case.

Comparison of the representative and consensus matrices for 38 taxa showed two essential differences. First, consensus had more ambiguous sites. That is, some nucleotides were ambiguous in consensus but not in representative (though the number of these was less than one percent of the total). Second, consensus had more total sequence. That is, some nucleotide positions were present in consensus but not in representative. Again, the size of the difference was small; there were less than 2000 such positions. Although both differences seem quantitatively minor, we felt that one or the other was likely to underlie the contrast in search difficulty. Therefore, we sought to isolate each of the two variables in turn – level of codon ambiguity, and total number of codons – and test the effect of each variable on search efficacy.

To test the effect of increased codon ambiguity, we built a modified representative matrix that differed from the original only in having a level of ambiguity comparable to that in the consensus matrix. We first aligned the consensus and representative amino acid sequences to each other, then converted each matrix back to nucleotide coding sequence. For each comparison of the same taxon between the two aligned matrices, we identified all of the amino acid positions at which there were multiple amino acids in the consensus matrix prior to reduction for phylogenetic analysis, which would result in ambiguity upon conversion to a phylogenetic data matrix by the consensus procedure. In those cases, we replaced the corresponding codon in the representative coding sequence with the consensus codon. The result was a matrix the same size as the original representative data matrix, with mostly the same sequence content, but with some ambiguity added. When this matrix was subjected to 100 ML searches, it returned the best topology far more often than did the unmodified representative matrix, and about as often as the consensus matrix. Thus, the increased ambiguity seemed to make tree search considerably easier.

To determine whether the greater total amount of sequence in the consensus matrix also affects search efficacy, we built a second modified representative matrix, intended to differ from the original in size but not level of ambiguity. To do this, we identified all the sites at which there was a gap character in the representative matrix and a non-gap character in the consensus matrix. We then added all of these additional non-gap characters to the representative matrix. Under ML search, this matrix behaved very much like the original representative matrix, finding the best tree only one or a few times in each 100 searches.

In summary, replacing non-ambiguous representative codons with ambiguous consensus codons essentially produced the consensus result, whereas adding the additional consensus data to the original representative matrix did not have the same transformative effect. We hypothesize that the small number of nucleotides that are ambiguous under consensus coding but not so under representative coding introduce conflicting signal in the latter matrix that makes finding the best tree topology more difficult than if these nucleotides are degenerated. The effect is idiosyncratic, in that we saw it only in the 38-taxon data set. We felt it was nonetheless worth investigating because essentially nothing was known about the comparative performance of these two procedures.
